# Systemic Inflammation and New-Onset Atrial Fibrillation in Acute Coronary Syndromes: A Single-Center Cohort Study

**DOI:** 10.3390/jcm15218528

**Published:** 2026-11-02

**Authors:** Ruxandra-Maria Băghină, Simina Crișan, Silvia Luca, Oana Pătru, Cristina-Maria Roman, Marian Morenci, Ionuț Mădălin Munteanu, Cristina Văcărescu, Constantin-Tudor Luca, Dan Gaiță

**Affiliations:** 1 Cardiology Department, “Victor Babes” University of Medicine and Pharmacy, 2 Eftimie Murgu Sq., 300041 Timisoara, Romania; ruxandra.croicu@umft.ro (R.-M.B.); silvia.luca@umft.ro (S.L.); oana.patru@umft.ro (O.P.); morenci.marian@student.uoradea.ro (M.M.); cristina.vacarescu@umft.ro (C.V.); constantin.luca@umft.ro (C.-T.L.); dan.gaita@umft.ro (D.G.); 2 Institute of Cardiovascular Diseases Timisoara, 13A Gheorghe Adam Street, 300310 Timisoara, Romania; cristinaroman1318@gmail.ro; 3 Research Center of the Institute of Cardiovascular Diseases Timisoara, 13A Gheorghe Adam Street, 300310 Timisoara, Romania; 4 Doctoral School, “Victor Babes” University of Medicine and Pharmacy, 2 Eftimie Murgu Sq., 300041 Timisoara, Romania; 5 Doctoral School of Biomedical Sciences, Faculty of Medicine and Pharmacy, University of Oradea, 410087 Oradea, Romania

**Keywords:** inflammation, new-onset atrial fibrillation, c reactive protein

## Abstract

**Background/Objectives**: New-onset atrial fibrillation (NOAF) is a common complication of acute coronary syndrome (ACS) and is associated with adverse clinical outcomes. Although systemic inflammation has been implicated in the pathogenesis of NOAF, the contribution of different inflammatory biomarkers and their incremental value beyond established clinical factors remain incompletely understood. This study aimed to examine the association between inflammatory biomarkers and NOAF in patients hospitalized with ACS and to assess their contribution beyond established clinical risk factors. **Materials and Methods**: This prospective single-center cohort included 100 consecutive patients hospitalized with ACS and without a documented history of atrial fibrillation. Comprehensive demographic, clinical, laboratory, echocardiographic, and angiographic data were collected. Systemic inflammatory status was assessed using C-reactive protein (CRP), the neutrophil-to-lymphocyte ratio (NLR), the monocyte-to-lymphocyte ratio (MLR), and the systemic immune-inflammation index (SII). Univariable and multivariable logistic regression analyses were performed to identify factors associated with NOAF. The incremental contribution of inflammatory biomarkers to apparent in-sample model discrimination was explored using receiver operating characteristic (ROC) curve analysis and DeLong’s test. Sensitivity analyses were performed according to the timing of NOAF occurrence. **Results**: NOAF occurred in 33 of 100 patients (33%) during hospitalization. Patients who developed NOAF were older and exhibited greater left atrial enlargement, more extensive coronary artery disease, and higher inflammatory biomarker levels. In univariable analyses, CRP, NLR, and SII were significantly associated with NOAF. In exploratory multivariable analyses adjusted for clinically selected covariates, CRP remained associated with NOAF (adjusted OR 1.71, 95% CI 1.08–2.91; *p* = 0.031), whereas NLR, SII, NT-proBNP, and troponin did not retain statistically significant associations. CRP demonstrated the highest numerical improvement in model discrimination, although the increase in AUC compared with the baseline clinical model was not statistically significant. Sensitivity analyses confirmed the consistency of the association between CRP and NOAF. **Conclusions**: In this exploratory single-center cohort, CRP remained associated with NOAF after adjustment for clinically selected covariates, supporting further investigation of the relationship between systemic inflammation and NOAF development. These findings require confirmation in larger cohorts with internal and external validation.

## 1. Introduction

Acute coronary syndromes remain a major cause of cardiovascular morbidity and mortality worldwide despite considerable improvements in diagnostic and therapeutic strategies. In addition to myocardial injury and ischemic complications, ACS is often accompanied by cardiac arrhythmias that may adversely affect both short- and long-term prognosis [1]. Among these, new-onset atrial fibrillation represents one of the most common supraventricular arrhythmias encountered during hospitalization for ACS and has consistently been associated with increased risks of heart failure, stroke, recurrent ischemic events, prolonged hospital stay, and mortality [2,3].

The pathophysiological mechanisms underlying NOAF in ACS are complex and multifactorial. Acute ischemia, atrial pressure overload, autonomic nervous system activation, electrolyte disturbances, and ventricular dysfunction have all been implicated in the development of atrial arrhythmias during the acute phase of coronary disease [4]. However, increasing evidence suggests that systemic inflammation may play a central role in both the initiation and maintenance of atrial fibrillation [5]. The inflammatory response initiated by plaque rupture and myocardial necrosis extends beyond the coronary circulation, leading to activation of multiple cellular and molecular pathways that may promote atrial electrical instability and structural remodeling.

Inflammation has emerged as a key contributor to cardiovascular disease progression and adverse clinical outcomes. Experimental and clinical studies have demonstrated that inflammatory mediators can alter atrial electrophysiological properties, increase oxidative stress, and promote fibrotic remodeling, thereby creating a substrate favorable for atrial fibrillation. Furthermore, inflammatory activity appears to be particularly pronounced during ACS, raising the possibility that patients with a heightened inflammatory burden may be more susceptible to developing NOAF.

Several biomarkers have been proposed as indicators of systemic inflammation in cardiovascular disease. Among them, C-reactive protein remains the most widely investigated acute-phase reactant and has been associated with infarct severity, adverse cardiovascular events, and mortality in patients with ACS [6]. In recent years, inflammation-related hematological indices derived from routine blood counts, including the neutrophil-to-lymphocyte ratio and the systemic immune-inflammation index (SII), have also attracted attention as accessible markers reflecting the balance between inflammatory and immune responses [7,8]. These parameters have demonstrated prognostic value in various cardiovascular conditions, including coronary artery disease and atrial fibrillation.

Despite increasing interest in the inflammatory basis of atrial fibrillation, the relationship between systemic inflammatory burden and NOAF during ACS remains incompletely defined. Previous studies have produced heterogeneous results, and most investigations have relied on inflammatory measurements obtained only at hospital admission. Consequently, limited data are available regarding the evolution of inflammatory markers during hospitalization and their potential association with the timing of NOAF occurrence [9,10].

Considering the clinical impact of NOAF and the growing recognition of inflammation as a potential therapeutic target, identifying reliable inflammatory predictors may improve risk stratification in patients presenting with ACS. Therefore, the present single-center cohort study aimed to investigate the association between systemic inflammatory markers and the occurrence of NOAF in patients hospitalized with ACS. Particular focus was placed on the evaluation of CRP dynamics, comparing values obtained at admission and at the time of arrhythmia onset, alongside routinely available inflammatory indices such as NLR and SII [11].

## 2. Materials and Methods

### 2.1. Study Design

This prospective observational study included patients presenting with suspected acute coronary syndromes who were consecutively admitted to the Cardiology Department, Acute Coronary Care Unit of the Institute of Cardiovascular Diseases, Timișoara, Romania, between February 2024 and September 2025. Among the 116 patients screened, 100 met the eligibility criteria and formed the final study cohort. The remaining 16 patients were excluded due to active malignancy (*n* = 3), autoimmune or systemic inflammatory diseases (*n* = 2), acute infection or myopericarditis (*n* = 6), and incomplete clinical or laboratory data or lack of informed consent (*n* = 5). Patients with a previous history of atrial fibrillation or atrial flutter were excluded to allow the assessment of new-onset atrial fibrillation during hospitalization. Additional exclusion criteria included active malignancy, autoimmune or systemic inflammatory diseases, acute infection, myopericarditis, incomplete clinical or laboratory data, and lack of written informed consent.

The diagnosis of ACS was established according to the current ESC guidelines and the Fifth Universal Definition of Myocardial Infarction. Patients were subsequently classified into one of three clinical presentations: unstable angina, NSTEMI, or STEMI. Myocardial infarction was diagnosed based on evidence of acute myocardial injury in the presence of clinical evidence of acute myocardial ischemia. In patients with myocardial infarction, electrocardiographic criteria were used to distinguish between STEMI and NSTEMI [12].

### 2.2. Data Collection, Clinical Assessment and Laboratory Assessment

Demographic and clinical data were prospectively collected throughout hospitalization. The variables documented included age, sex, smoking status, arterial hypertension, diabetes mellitus, chronic kidney disease, dyslipidemia, body mass index (BMI), and heart rate and systolic and diastolic blood pressure at admission. Arterial hypertension was defined as an office systolic blood pressure ≥140 mmHg and/or diastolic blood pressure ≥90 mmHg, or the use of antihypertensive medication, in accordance with the 2024 ESC Guidelines for the management of elevated blood pressure and hypertension [13,14]. Chronic kidney disease was defined as an estimated glomerular filtration rate (eGFR) < 60 mL/min/1.73 m^2^ persisting for at least 3 months, in accordance with the 2024 KDIGO guidelines [15]. Patients actively smoking at the time of admission were classified as current smokers, while former and never-smokers were considered non-current smokers.

New-onset atrial fibrillation was defined as the first documented episode of AF occurring during the index hospitalization in patients without a previous history of AF. The diagnosis was established according to the 2024 ESC Guidelines for the management of atrial fibrillation and required ECG documentation of a rhythm characterized by irregular R–R intervals and the absence of distinct P waves, recorded on a 12-lead ECG or for at least 30 s on an ECG rhythm strip [16]. All patients were in sinus rhythm on the admission 12-lead ECG and were initially admitted to the Acute Coronary Care Unit, where continuous cardiac rhythm monitoring was performed using bedside ECG monitors. The duration of monitoring in this unit varied according to the patients’ clinical condition and hemodynamic stability. Following clinical stabilization and transfer to the Cardiology Department, patients in whom atrial fibrillation had not been detected during their coronary care unit stay underwent 24-h ambulatory ECG monitoring using either a BTL Holter monitoring system (BTL Industries Ltd., Hertfordshire, UK) or a TLC9803 Holter recorder (Contec Medical Systems Co., Ltd., Qinhuangdao, China). Holter recordings were systematically examined for the detection of new-onset atrial fibrillation. The timing of Holter monitoring varied according to the availability of recording devices, as no predefined day of hospitalization was established for its initiation. Episodes of NOAF were identified through continuous bedside ECG monitoring during the coronary care unit stay and through ECG Holter recordings following transfer to the cardiology ward. The median duration of hospitalization was 6 days (interquartile range: 4–9 days).

Routine laboratory testing was performed as part of standard clinical care and included fasting plasma glucose; serum creatinine; total leukocyte count; absolute neutrophil, lymphocyte, and monocyte counts; and platelet count. Cardiac biomarkers, including cardiac troponin I and N-terminal pro-B-type natriuretic peptide (NT-proBNP), were measured at hospital admission. The reported biomarker values represent admission measurements rather than peak concentrations during hospitalization. Renal function was assessed using serum creatinine and estimated glomerular filtration rate (eGFR), expressed as mL/min/1.73 m^2^, with eGFR values derived using the MedCalc online calculator (accessed in October 2025).

The inflammatory profile was assessed using C-reactive protein and composite indices derived from peripheral blood cell counts, including the neutrophil-to-lymphocyte ratio, platelet-to-lymphocyte ratio (PLR), monocyte-to-lymphocyte ratio, and systemic immune-inflammation index. NLR, PLR, and MLR were calculated by dividing the absolute neutrophil, platelet, and monocyte counts, respectively, by the absolute lymphocyte count. Because blood cell counts were recorded in cells/µL, SII was calculated as (platelet count × neutrophil count/lymphocyte count)/1000 and was reported in ×10^9^/L, corresponding to the conventional scale used in the literature. In patients who developed NOAF, CRP was also measured from a separate blood sample obtained on the day of the first documented arrhythmic episode. Therefore, two distinct CRP measurements were available for all 33 patients with NOAF, including those in whom the arrhythmia occurred within the first 24 h of hospitalization [17].

### 2.3. Baseline Cardiac Assessment

A comprehensive cardiovascular evaluation was performed at hospital admission, including a standard 12-lead electrocardiogram (ECG), followed by a transthoracic echocardiographic examination performed according to standard echocardiographic protocols, by using a Vivid IQ ultrasound system (GE Healthcare, Milwaukee, WI, USA) equipped with a 2.5 MHz phased-array transducer. Left ventricular ejection fraction (LVEF) was calculated using the biplane Simpson method from apical four- and two-chamber views. Left atrial (LA) anteroposterior diameter was measured at end-systole in the parasternal long-axis view. LA volume was determined at end-systole from apical four- and two-chamber views using the biplane method of disks [18].

### 2.4. Coronary Angiographic Evaluation

Invasive coronary angiography was performed in all study participants as part of routine ACS management. The vascular access site (radial or femoral) was selected at the discretion of the treating interventional cardiologist. Coronary anatomy and the extent and distribution of coronary artery disease were assessed using standard angiographic projections. Revascularization strategy, including percutaneous coronary intervention (PCI), when indicated, was determined according to angiographic characteristics and the patient’s clinical condition, following current clinical practice guidelines.

### 2.5. Study Endpoints

The primary study endpoint was the occurrence of new-onset atrial fibrillation during index hospitalization. Patients were categorized into two groups according to the presence or absence of NOAF.

Secondary endpoints included the timing of NOAF onset (<24 h, 24–48 h, and >48 h after admission), the association between inflammatory biomarkers and NOAF occurrence, temporal changes in C-reactive protein levels among patients who developed NOAF, and the identification of independent clinical, laboratory, echocardiographic, and angiographic predictors of NOAF. The incremental predictive value of inflammatory biomarkers beyond established clinical risk factors was also assessed.

### 2.6. Ethical Considerations

The study was carried out in accordance with the ethical principles outlined in the Declaration of Helsinki. Ethical approval was obtained from the Institutional Ethics Committee of the “Victor Babeș” University of Medicine and Pharmacy Timișoara (approval No. 54, 2 October 2023) and the Institute of Cardiovascular Diseases Timișoara (approval No. 438, 22 January 2024). Written informed consent was obtained from all participants before enrollment.

### 2.7. Statistical Analysis

Statistical analyses were performed using R software version 4.5.0 (R Foundation for Statistical Computing, Vienna, Austria) within the RStudio environment (version 2025.5.0.496). A two-sided *p* value < 0.05 was considered statistically significant.

Continuous variables are summarized as mean ± standard deviation (SD) or median with interquartile range (IQR) according to their distribution, while categorical variables are reported as frequencies and percentages. Group comparisons were performed using the independent-samples *t* test or the Mann–Whitney *U* test for continuous variables and the χ^2^ test or Fisher’s exact test for categorical variables, as appropriate.

Admission inflammatory status was assessed using CRP, leukocyte, neutrophil, lymphocyte, monocyte, and platelet counts, together with the inflammatory indices NLR, PLR, MLR, and SII. CRP, NT-proBNP, troponin, NLR, and SII expressed in ×10^9^/L showed right-skewed distributions and were therefore log1p-transformed before being entered into the regression models.

Among patients who developed NOAF, changes in CRP over time were assessed by comparing the admission measurement with a second measurement obtained from a separate blood sample on the day of the first documented arrhythmic episode. CRP delta (ΔCRP) was calculated as the difference between CRP measured on the day of NOAF onset and CRP measured at hospital admission. Two distinct blood samples were available for all 33 patients with NOAF, including the 22 patients who developed NOAF within the first 24 h. In every early-onset case, the admission blood sample was obtained before the first documented AF episode. Among these 22 patients, the two separate samples yielded the same reported CRP concentration in 18 patients and different concentrations in four patients; consequently, ΔCRP was 0 in 18 patients because no numerical change was observed, rather than because the admission value was reused or imputed. Paired CRP values were compared using the Wilcoxon signed-rank test. Differences in ΔCRP according to NOAF timing and procedural status were assessed using the Kruskal–Wallis and Mann–Whitney U tests, respectively. A sensitivity analysis was additionally restricted to the 11 patients who developed NOAF more than 24 h after admission, in whom the interval between the two CRP measurements was longer than 24 h.

Factors associated with NOAF were initially explored using univariable logistic regression. The comparison between patients with and without NOAF and the evaluation of admission inflammatory biomarkers were defined prospectively; however, the detailed regression modeling strategy was not formally preregistered and should therefore be considered exploratory. Five baseline covariates—age, male sex, left ventricular ejection fraction, serum creatinine, and STEMI presentation—were selected before multivariable model fitting on clinical grounds to represent demographic characteristics, ventricular function, renal function, and ACS phenotype. These covariates were entered simultaneously into the baseline clinical model, and no automated or stepwise variable-selection procedure was used. To limit the number of parameters relative to the 33 NOAF events, CRP, NLR, and SII were selected for the adjusted biomarker comparison because they had prior published relevance in ACS and NOAF and showed statistically significant associations in the univariable logistic analyses. Each marker was evaluated separately by adding it to the common baseline clinical model. MLR was evaluated descriptively and in the univariable analysis but was not carried forward into the adjusted comparison because its univariable logistic association was not statistically significant and it represented an additional leukocyte-derived ratio with overlapping biological information. A separate model was developed to evaluate the associations of NT-proBNP and troponin with NOAF. Results are reported as odds ratios (ORs) with 95% confidence intervals (CIs), while model fit was assessed using the Akaike information criterion (AIC).

To examine potential confounding by atrial remodeling, an additional parsimonious sensitivity model was fitted that included admission CRP, age, and a single measure of left atrial size. Left atrial volume was selected rather than left atrial diameter to avoid including two related echocardiographic measures in the same model and because volume provides a more comprehensive measure of atrial size. CRP was log1p-transformed, and left atrial volume was expressed per 10 mL increase. The sensitivity model was deliberately restricted to three predictors because of the limited number of NOAF events. This atrial-remodeling sensitivity analysis was conducted post hoc and should be interpreted as exploratory.

Model discrimination was assessed by receiver operating characteristic (ROC) curve analysis, and the area under the curve (AUC) was calculated for each model. Differences between AUCs were assessed using DeLong’s test on a common complete-case dataset. Because model discrimination was assessed in the same cohort used for model estimation, and no bootstrap or other resampling-based internal validation was performed, the reported AUC values represent apparent in-sample performance and may be optimistic. No external validation was performed. These analyses were intended to assess associations and apparent incremental discrimination rather than to develop or validate a clinical prediction model.

Sensitivity analyses included separate logistic regression models for early (<24 h) and delayed (>24 h) NOAF. For the timing-based sensitivity analyses, Wald 95% confidence intervals and two-sided Wald *p* values were calculated from the same model coefficients and standard errors to ensure internally consistent inference. All statistical analyses were conducted using complete-case data. Variables with sparse data were not entered into logistic regression analyses because of the expected instability of the corresponding effect estimates.

## 3. Results

Among the 116 patients initially evaluated for suspected acute coronary syndrome, 100 fulfilled the eligibility criteria and were included in the final study cohort. The study population comprised 81 patients with STEMI, 12 with NSTEMI, and 7 with unstable angina. New-onset atrial fibrillation occurred in 33 patients (33.0%) during hospitalization. Among these, 22 patients (66.7%) developed NOAF within the first 24 h, 7 (21.2%) between 24 and 48 h, and 4 (12.1%) more than 48 h after admission.

### 3.1. Incidence and In-Hospital Characteristics of New-Onset Atrial Fibrillation

**Figure 1:**
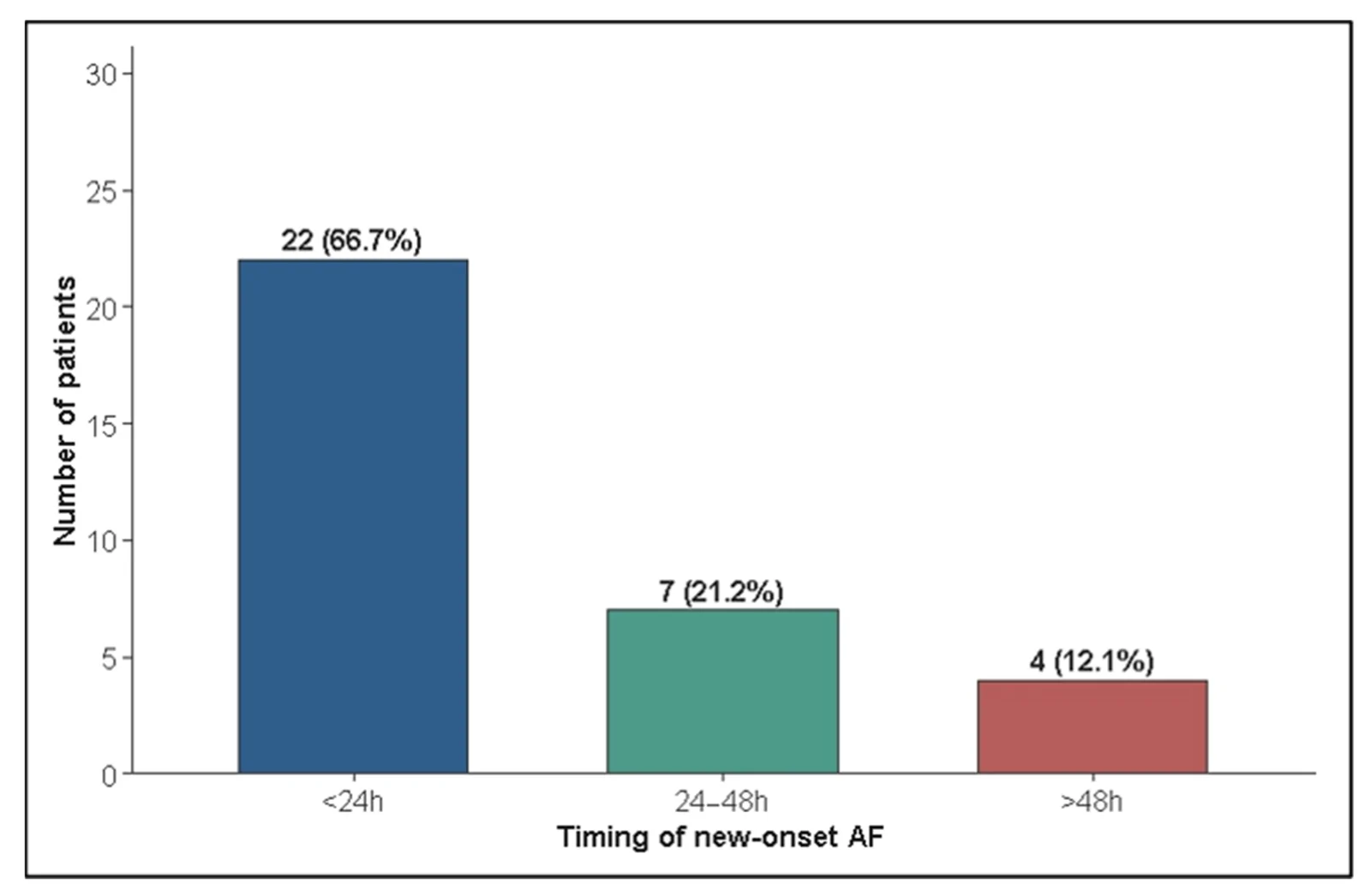
Distribution of new-onset AF according to timing of onset. Data are presented as number and percentage of patients among those who developed new-onset AF.

The occurrence of new-onset atrial fibrillation during hospitalization was evaluated in the entire study cohort. During hospitalization, 33 patients (33.0%) experienced NOAF, while the remaining 67 patients (67.0%) had no documented atrial fibrillation. Among patients with NOAF, 22 (66.7%) developed arrhythmia within the first 24 h after admission, while 7 (21.2%) developed NOAF between 24 and 48 h and 4 (12.1%) after 48 h (Table 1, Figure 1). Regarding the temporal relationship with coronary intervention, 22 episodes (66.7%) occurred following the procedure, whereas 11 (33.3%) occurred before coronary intervention. The median ventricular rate during NOAF was 130 bpm (IQR, 120–140 bpm). NOAF episodes were managed according to the patients’ clinical status, with antiarrhythmic therapy and beta-blockers administered as clinically indicated. Conversion to sinus rhythm before hospital discharge occurred in 30 patients (90.9%), while three patients (9.1%) remained in atrial fibrillation.

### 3.2. Baseline Clinical, Echocardiographic, and Biochemical Characteristics According to New-Onset Atrial Fibrillation

Baseline demographic, clinical, echocardiographic, and biochemical characteristics according to NOAF status are summarized in Table 2. Patients who developed NOAF were significantly older than those without documented atrial fibrillation (72 [65–77] vs. 60 [52–72] years, *p* < 0.001) and had a higher body mass index (29.0 [26.1–32.7] vs. 27.3 [25.5–29.2] kg/m^2^, *p* = 0.033). Hypertension (87.9% vs. 62.7%, *p* = 0.009), dyslipidemia (93.9% vs. 58.2%, *p* < 0.001), and chronic kidney disease (36.4% vs. 7.5%, *p* < 0.001), were more frequent in the NOAF group. No significant between-group differences were observed in the proportion of male patients, diabetes mellitus, or current smoking status.

Admission systolic blood pressure, diastolic blood pressure, and heart rate were comparable between groups. Left atrial diameter (4.3 [4.2–4.6] vs. 3.8 [3.2–4.1] cm, *p* < 0.001) and left atrial volume (87 [72–100] vs. 59 [46.5–75.5] mL, p < 0.001), both measured at hospital admission, were significantly greater in patients who subsequently developed NOAF. Left ventricular ejection fraction was similar between groups (35 [30–40] % vs. 40 [35–45] %, *p* = 0.167).

### 3.3. Acute Coronary Syndrome Phenotype According to New-Onset Atrial Fibrillation

The distribution of ACS phenotype according to NOAF status is presented in Table 3. No significant differences were observed in ACS presentation between groups (*p* = 0.851). STEMI was the predominant phenotype in both patients with and without NOAF (81.8% vs. 80.6%), whereas the proportions of NSTEMI and unstable angina were similar. Overall, ACS phenotype was not associated with the occurrence of NOAF.

### 3.4. Coronary Angiographic and Procedural Characteristics According to New-Onset Atrial Fibrillation

Coronary angiographic and procedural characteristics were subsequently compared according to NOAF status to evaluate potential differences in coronary artery disease burden and revascularization patterns (Table 4). The overall distribution of coronary artery disease extent differed significantly between groups (*p* < 0.001). Patients who developed NOAF had a markedly higher prevalence of three-vessel coronary artery disease than those without NOAF (48.5% vs. 13.4%, *p* < 0.001).

The prevalence of LAD and LCx involvement was similar between groups, while RCA involvement was more frequent among patients with NOAF, although the difference did not reach statistical significance (69.7% vs. 49.3%, *p* = 0.053). Conversely, left main coronary artery involvement was significantly more frequent among patients who developed NOAF (12.1% vs. 1.5%, *p* = 0.040).

PCI was performed in nearly all patients, with comparable rates between the NOAF and non-NOAF groups (97.0% vs. 98.5%, *p* = 1.000). Complete revascularization was less frequent among patients who developed NOAF (54.5% vs. 74.6%), whereas incomplete revascularization was more frequent (45.5% vs. 25.4%). However, the difference in revascularization status between groups was not statistically significant (*p* = 0.067).

Overall, patients who developed NOAF had more extensive coronary artery disease, characterized primarily by a higher prevalence of three-vessel disease and more frequent left main coronary artery involvement.

### 3.5. Admission Cardiac Biomarkers According to New-Onset Atrial Fibrillation

**Figure 2:**
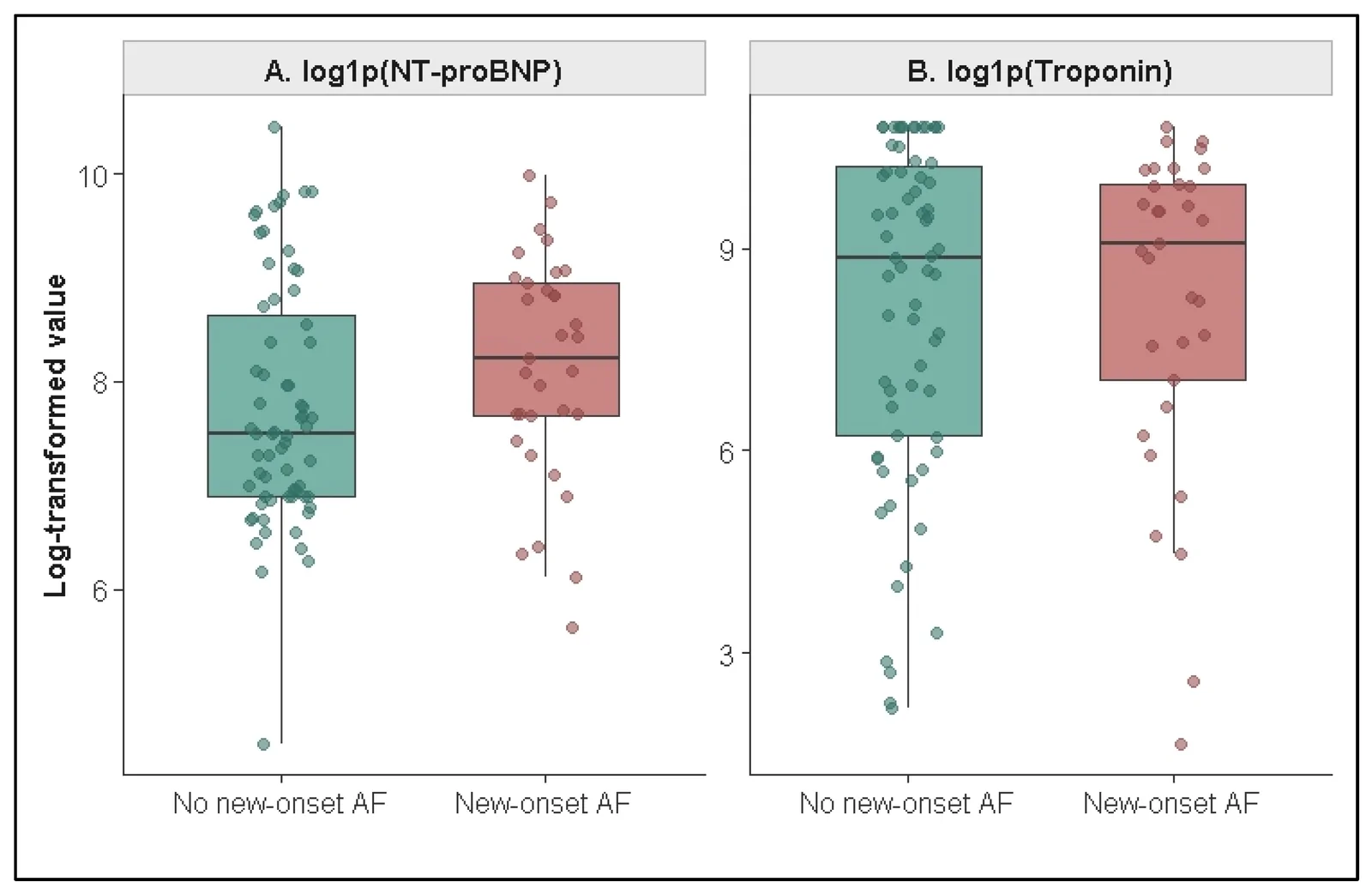
Distribution of log-transformed NT-proBNP and troponin values according to new-onset atrial fibrillation status. Boxplots illustrate the distribution of log-transformed admission NT-proBNP and troponin values in patients with and without NOAF. NT-proBNP concentrations were numerically higher in patients who subsequently developed NOAF, whereas troponin concentrations were comparable between groups. Abbreviations: NOAF, new-onset atrial fibrillation; NT-proBNP, N-terminal pro-B-type natriuretic peptide.

The relationship between admission cardiac biomarkers and the subsequent occurrence of NOAF was assessed using troponin and NT-proBNP concentrations (Table 5 and Figure 2). Admission troponin concentrations were similar in patients who developed NOAF and those who did not (8673 [1146–20,774] vs. 7000 [501–27,347.5], *p* = 0.965). In contrast, admission NT-proBNP concentrations were numerically higher among patients who developed NOAF (3750 [2134.4–7650] vs. 1800.4 [988.8–5622.9] pg/mL), although the difference did not reach statistical significance (*p* = 0.067). Figure 2 illustrates the distribution of log-transformed NT-proBNP values, showing a trend toward higher concentrations in the NOAF group despite substantial overlap between groups. Overall, admission troponin levels were comparable between groups, whereas NT-proBNP showed a nonsignificant tendency toward higher concentrations in patients who developed NOAF.

### 3.6. Admission Inflammatory Profile According to New-Onset Atrial Fibrillation

**Figure 3:**
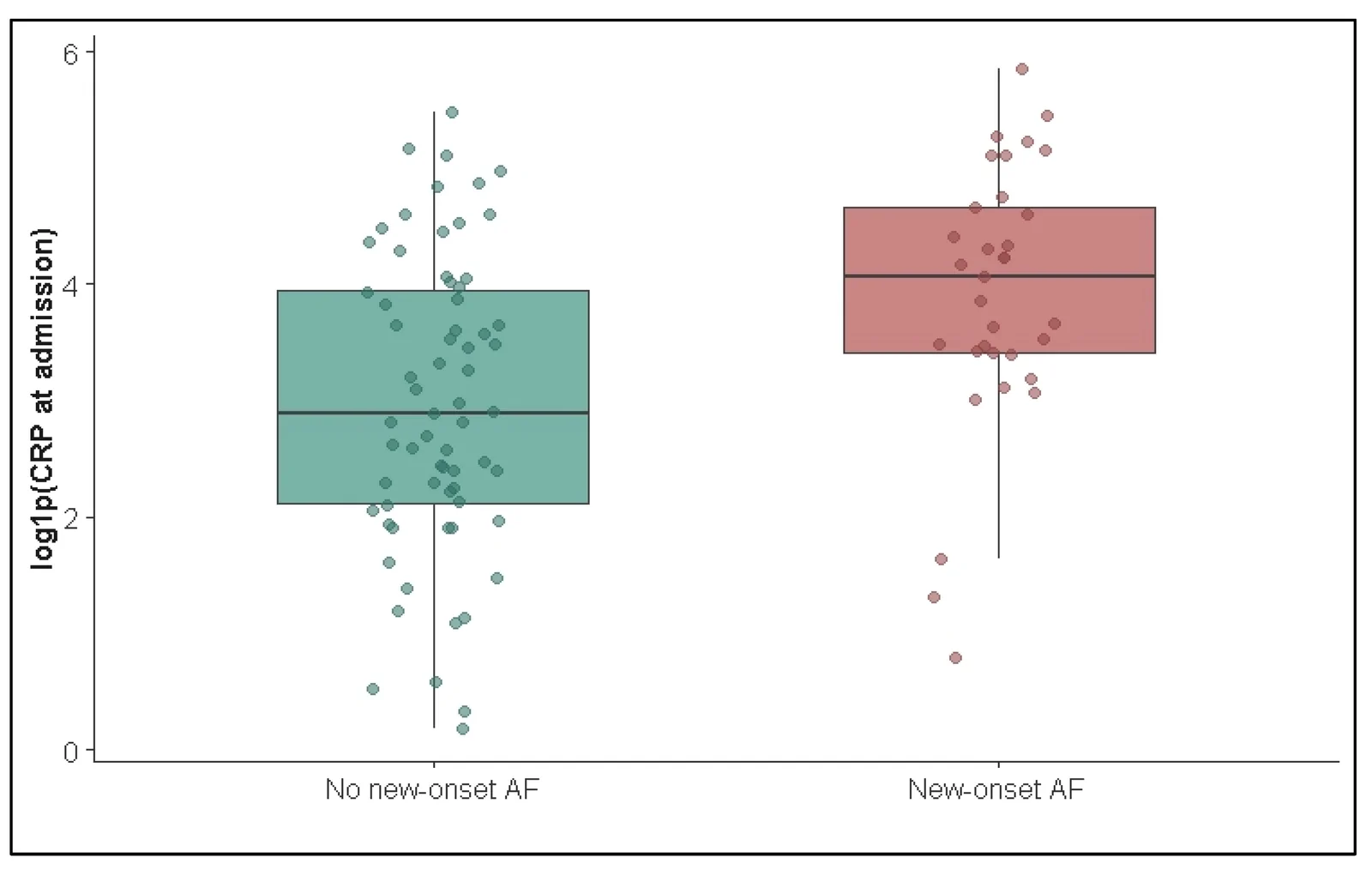
Admission C-reactive protein according to new-onset atrial fibrillation. Boxplot of log-transformed admission C-reactive protein values in patients with and without new-onset atrial fibrillation. Individual dots represent individual patients. CRP values were log-transformed using the log1p function.

**Figure 4:**
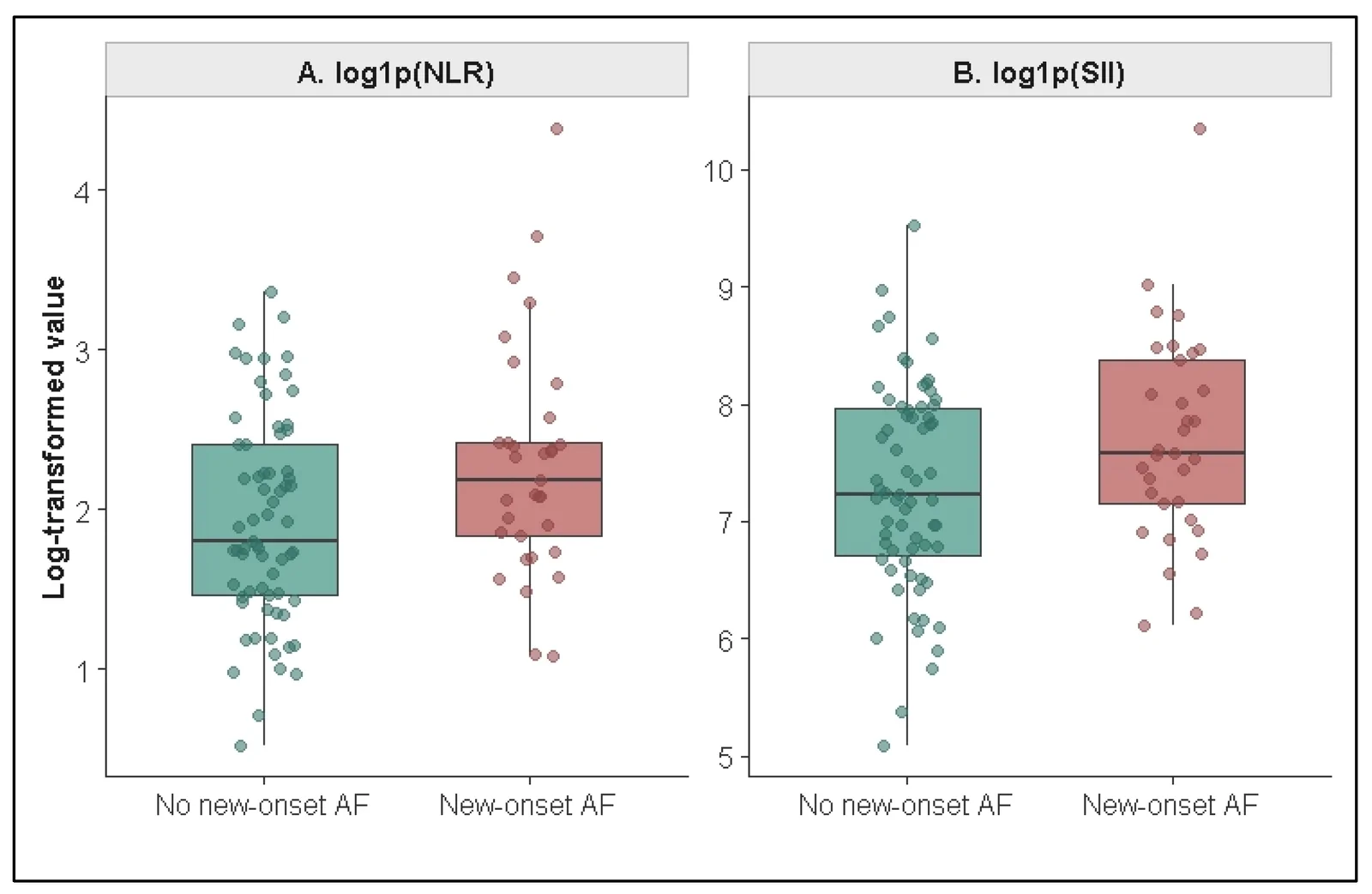
Admission composite inflammatory indices according to new-onset atrial fibrillation.

Given the central role of inflammation in the present study, inflammatory biomarkers measured at hospital admission were compared according to subsequent NOAF status (Table 6; Figure 3 and Figure 4). Patients who subsequently developed NOAF had significantly higher admission CRP concentrations than those without documented atrial fibrillation (57.2 [29.4–105.3] vs. 16.9 [7.3–51.1] mg/L, *p* < 0.001). This difference remained evident after log-transformation (Figure 3). Although total leukocyte and neutrophil counts were comparable between groups, patients who developed NOAF had lower lymphocyte counts (1250 [940–1930] vs. 1680 [1200–2315] cells/µL, *p* = 0.028), whereas monocyte and platelet counts did not differ significantly.

Composite inflammatory indices were significantly higher in patients who subsequently developed NOAF. The neutrophil-to-lymphocyte ratio (NLR) (7.87 [5.23–10.23] vs. 5.01 [3.29–10.06], *p* = 0.036), monocyte-to-lymphocyte ratio (MLR) (0.60 [0.35–0.84] vs. 0.38 [0.26–0.58], *p* = 0.008), and systemic immune-inflammation index (SII) (1972.6 [1276.9–4327.0] vs. 1371.9 [823.0–2863.4] ×10^9^/L, *p* = 0.040) were all significantly higher in the NOAF group. Although the platelet-to-lymphocyte ratio was numerically higher in patients with NOAF, the difference did not reach statistical significance (196.8 [137.3–330.3] vs. 152.3 [110.0–213.4], *p* = 0.053). The distribution of log-transformed NLR and SII values is shown in Figure 4. Overall, patients who subsequently developed NOAF were characterized by a more pronounced inflammatory profile at hospital admission, including higher CRP levels, lower lymphocyte counts, and significantly higher NLR, MLR, and SII values.

Boxplots of log-transformed NLR and SII values in patients with and without NOAF. Individual dots represent individual patients. Values were log-transformed using the log1p function. SII was calculated and expressed in ×10^9^/L before transformation.

### 3.7. Temporal Changes in C-Reactive Protein in Patients with New-Onset Atrial Fibrillation

**Figure 5:**
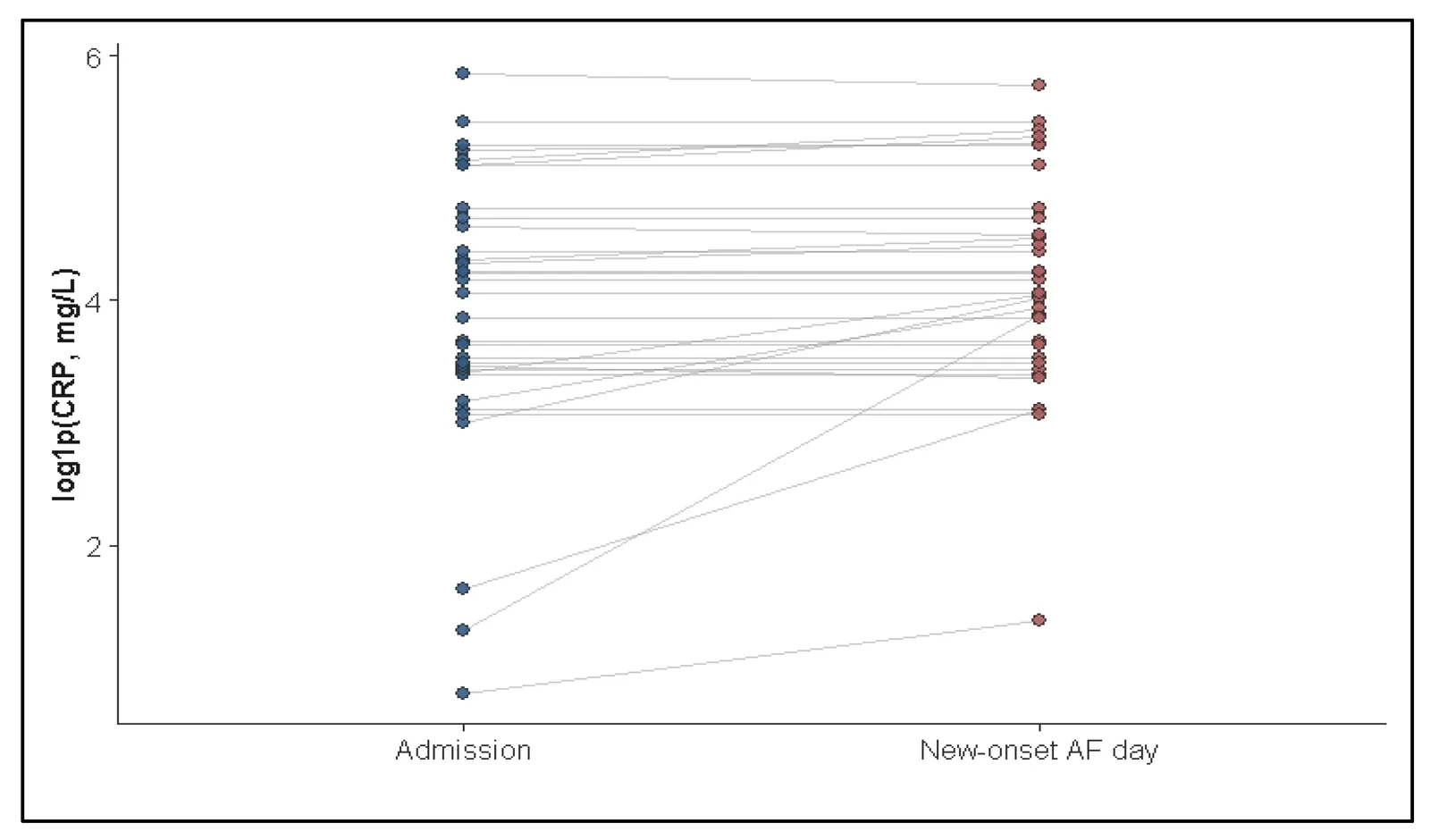
Individual C-reactive protein trajectories from hospital admission to new-onset atrial fibrillation. Each line connects CRP concentrations obtained from two separate blood samples for an individual patient: the admission sample and the sample obtained on the day of the first documented NOAF episode. Among the 22 patients with NOAF onset within 24 h, the admission sample preceded the arrhythmic episode in every case, and the two samples yielded the same reported CRP concentration in 18 patients. CRP values were log-transformed using the log1p function to improve visualization. Abbreviations: CRP, C-reactive protein; NOAF, new-onset atrial fibrillation.

Changes in CRP concentrations between hospital admission and the onset of NOAF were evaluated in all 33 patients who developed NOAF, each of whom had two measurements obtained from separate blood samples (Table 7; Figure 5). Median CRP concentrations were elevated at admission (57.2 [29.4–105.3] mg/L), with a value of 57.2 [33.0–105.3] mg/L on the day of NOAF onset. Paired within-patient analysis demonstrated a significant change between the two measurements (Wilcoxon signed-rank test, *p* = 0.020), with a median CRP delta of 0 [0–12.3] mg/L.

Among the 22 patients who developed NOAF within the first 24 h, the admission and NOAF-day CRP values were obtained from separate blood samples, and the admission sample preceded the first documented AF episode in every case. The two samples yielded the same reported CRP concentration in 18 patients (81.8%) and different concentrations in four patients (18.2%), resulting in a median CRP delta of 0 [0–0] mg/L. Median CRP delta was 15.0 [5.7–27.0] mg/L in patients with NOAF onset between 24 and 48 h and 28.6 [7.5–46.0] mg/L in those with onset after 48 h. Median CRP delta was numerically larger in the later-onset categories; however, the overall difference among timing groups was not statistically significant (Kruskal–Wallis test, *p* = 0.064).

CRP dynamics did not differ according to procedural status. Patients with anteprocedural NOAF had a median CRP delta of 0 [0–0] mg/L, whereas those with postprocedural NOAF had a median CRP delta of 0 [0–14.3] mg/L (*p* = 0.163). Individual CRP trajectories are presented in Figure 5.

A sensitivity analysis was restricted to the 11 patients who developed NOAF more than 24 h after admission and therefore had a sampling interval longer than 24 h between the admission and NOAF-day measurements. In this subgroup, CRP increased from 72.7 [26.3–134.7] mg/L at admission to 85.0 [52.7–144.5] mg/L on the day of NOAF onset (Wilcoxon signed-rank *p* = 0.049), with a median ΔCRP of 15.0 [5.7–31.6] mg/L. Given the small size of this subgroup and the variability in sampling intervals, this finding should be considered exploratory and does not establish a temporal or causal relationship between increasing CRP concentrations and NOAF onset.

### 3.8. Univariable Predictors of New-Onset Atrial Fibrillation

Univariable logistic regression analyses were performed to identify variables associated with the occurrence of new-onset atrial fibrillation (NOAF) during hospitalization. Candidate predictors included demographic, clinical, echocardiographic, biochemical, inflammatory, cardiac biomarker, ACS phenotype, and angiographic variables. The results are summarized in Table 8.

Older age was significantly associated with higher odds of NOAF, with an 8% increase in odds for each additional year of age (OR 1.08, 95% CI 1.04–1.13, *p* < 0.001). Higher body mass index was also associated with increased odds of NOAF (OR 1.17, 95% CI 1.04–1.33, *p* = 0.013). Among cardiovascular comorbidities, hypertension, dyslipidemia, and chronic kidney disease were significantly associated with NOAF, with particularly strong associations observed for dyslipidemia (OR 11.13, 95% CI 3.02–72.28, *p* = 0.002) and chronic kidney disease (OR 7.09, 95% CI 2.34–24.52, *p* < 0.001). Male sex was not significantly associated with NOAF.

Echocardiographic measures of left atrial size were strongly associated with NOAF. Greater left atrial diameter (OR 10.03 per cm increase, 95% CI 3.74–33.53, *p* < 0.001) and left atrial volume (OR 1.04 per mL increase, 95% CI 1.02–1.07, *p* < 0.001) were associated with higher odds of NOAF, whereas left ventricular ejection fraction showed no significant association.

Regarding biochemical variables, creatinine showed a borderline association with higher odds of NOAF (OR 1.71, 95% CI 0.97–3.15, *p* = 0.069).

Among inflammatory biomarkers, higher log-transformed CRP was associated with greater odds of NOAF (OR 1.91, 95% CI 1.31–2.91, *p* = 0.001). Similar associations were observed for log-transformed NLR (OR 2.13, 95% CI 1.14–4.19, *p* = 0.021) and SII (OR 1.74, 95% CI 1.07–2.95, *p* = 0.031), whereas MLR showed a non-significant trend (OR 1.77, 95% CI 0.96–3.79, *p* = 0.093). Although MLR differed significantly between groups in the rank-based comparison presented in Table 6, it was not significantly associated with NOAF in the univariable logistic model. Because these analyses evaluate different aspects of the data and MLR did not meet the stated criterion for inclusion in the adjusted biomarker comparison, it was not examined further in the multivariable models.

Cardiac biomarkers and ACS phenotype were also evaluated in relation to NOAF. NT-proBNP showed a positive trend toward higher odds of NOAF (OR 1.35, 95% CI 0.93–1.98, *p* = 0.118), although statistical significance was not reached. Troponin showed no significant association with NOAF (OR 1.02, 95% CI 0.86–1.22, *p* = 0.843). Similarly, STEMI presentation was not significantly associated with NOAF (OR 0.81, 95% CI 0.29–2.40, *p* = 0.718).

Angiographic analysis showed a strong association between three-vessel coronary artery disease and NOAF (OR 6.07 (95% CI 2.29–16.09, *p* < 0.001), while right coronary artery involvement showed a borderline association (OR 2.37, 95% CI 1.00–5.92, *p* = 0.056).

### 3.9. Multivariable Logistic Regression Models for New-Onset Atrial Fibrillation

A baseline multivariable logistic regression model was constructed to evaluate factors associated with new-onset atrial fibrillation after adjustment for relevant clinical covariates (Table 9). The model included age, male sex, left ventricular ejection fraction, creatinine, and STEMI presentation. Within this exploratory model, age remained associated with NOAF, with an 8% increase in the odds of NOAF for each additional year of age (adjusted OR 1.08, 95% CI 1.03–1.14, *p* = 0.003). Male sex, left ventricular ejection fraction, creatinine, and STEMI presentation did not show significant associations with NOAF in the adjusted model.

Clinical models incorporating inflammatory biomarkers are presented in Table 10. Each biomarker was added separately to the baseline clinical model. Within this exploratory multivariable analysis, log-transformed CRP remained associated with NOAF after adjustment for the baseline clinical covariates (adjusted OR 1.71, 95% CI 1.08–2.91, *p* = 0.031). The clinical + CRP model had the lowest AIC among the fitted biomarker-extended models (AIC = 118.5), although this comparison should be interpreted cautiously given the limited number of events. Log-transformed NLR and SII did not retain statistically significant associations with NOAF after adjustment. The clinical + NLR model yielded an adjusted OR of 1.75 (95% CI 0.84–3.92, *p* = 0.150; AIC = 121.6), while the clinical + SII model yielded an adjusted OR of 1.52 (95% CI 0.84–2.88, *p* = 0.176; AIC = 121.9).

To further evaluate the association of biomarkers reflecting myocardial injury and hemodynamic stress with NOAF, a multivariable model including troponin and NT-proBNP was constructed (Table 11). Within this exploratory model, age remained associated with NOAF (adjusted OR 1.08, 95% CI 1.04–1.14, *p* < 0.001), whereas male sex, left ventricular ejection fraction, log-transformed NT-proBNP, and log-transformed troponin were not significantly associated with NOAF after adjustment. NT-proBNP showed an adjusted OR of 1.17 (95% CI 0.73–1.89, *p* = 0.504), while troponin showed an adjusted OR of 0.99 (95% CI 0.81–1.23, *p* = 0.951).

### 3.10. Discriminative Performance of the Clinical and Biomarker-Extended Models

**Figure 6:**
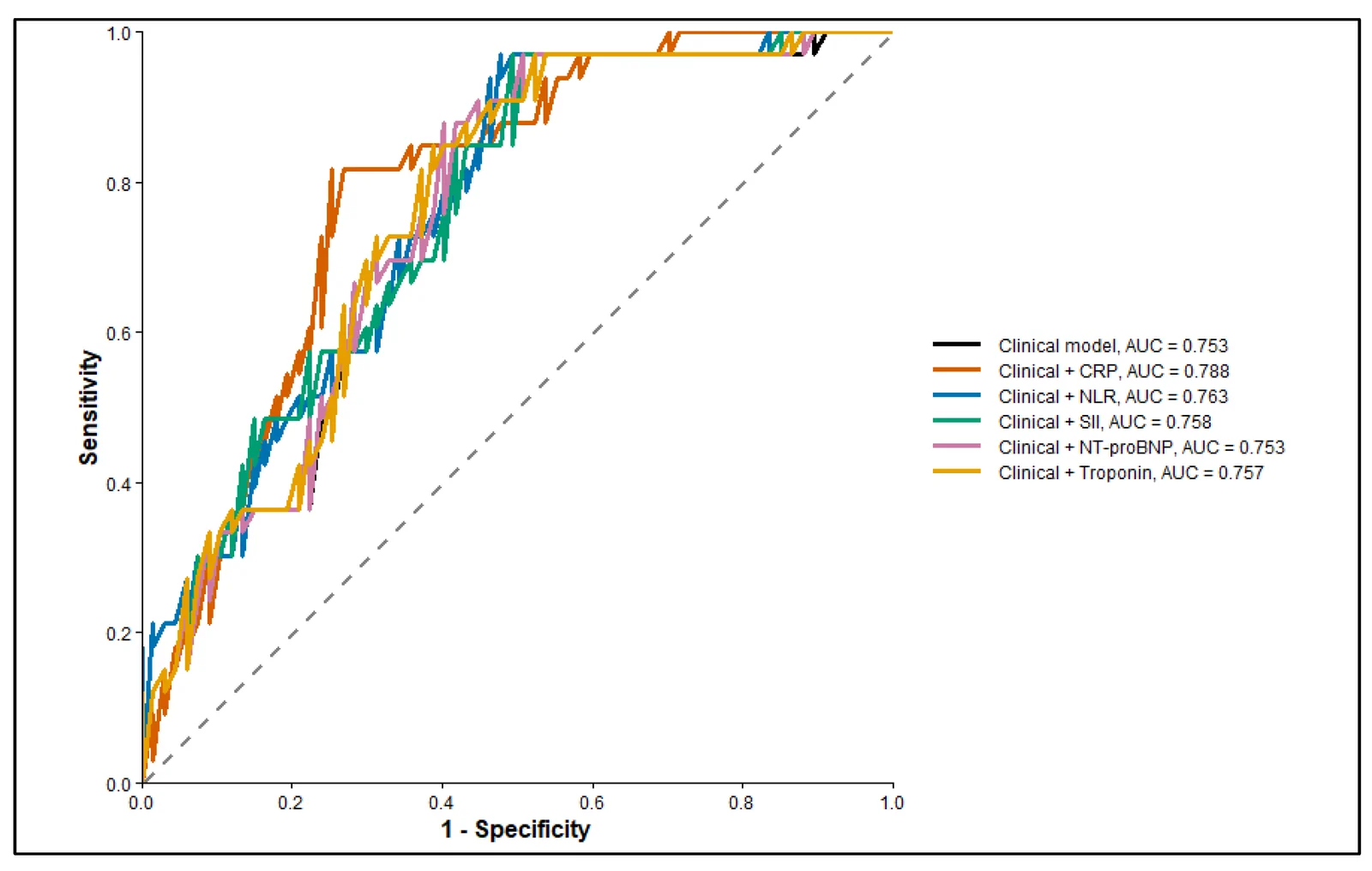
Receiver operating characteristic (ROC) curves of the baseline clinical model and biomarker-extended models for predicting new-onset atrial fibrillation. The baseline clinical model included age, male sex, left ventricular ejection fraction, creatinine, and STEMI. Separate models were constructed by adding CRP, NLR, SII, NT-proBNP, or troponin to the baseline clinical model. Area under the curve (AUC) values are displayed in the legend, and comparisons with the baseline clinical model were performed using DeLong’s test. The dashed diagonal line represents the line of no discrimination (AUC = 0.50), corresponding to prediction by chance. Abbreviations: AUC, area under the receiver operating characteristic curve; CRP, C-reactive protein; LVEF, left ventricular ejection fraction; NLR, neutrophil-to-lymphocyte ratio; NT-proBNP, N-terminal pro-B-type natriuretic peptide; ROC, receiver operating characteristic; SII, systemic immune-inflammation index; STEMI, ST-segment elevation myocardial infarction.

The performance of the baseline clinical model and biomarker-extended models is summarized in Table 12 and illustrated in Figure 6. The baseline clinical model, including age, male sex, left ventricular ejection fraction, creatinine, and STEMI presentation, yielded an apparent AUC of 0.753 (95% CI 0.656–0.849), reflecting moderate in-sample discrimination.

Among the evaluated biomarkers, the addition of log-transformed CRP resulted in the highest AUC, increasing it from 0.753 to 0.788 (95% CI 0.698–0.877). However, this increase was not statistically significant compared with the baseline clinical model according to DeLong’s test (*p* = 0.266).

The addition of log-transformed NLR and SII resulted in only minor changes in model performance, with AUCs of 0.763 (95% CI 0.668–0.857; *p* = 0.537 vs. the baseline model) and 0.758 (95% CI 0.662–0.853; *p* = 0.765), respectively.

Similarly, the addition of cardiac biomarkers did not significantly improve model performance. The model incorporating log-transformed NT-proBNP yielded an AUC of 0.753 (95% CI 0.657–0.849; *p* = 0.685 vs. the baseline model), while the model incorporating log-transformed troponin yielded an AUC of 0.757 (95% CI 0.662–0.852; *p* = 0.403).

Overall, the baseline clinical model showed moderate apparent discrimination within the study cohort. Although CRP provided the greatest numerical increase in AUC among the evaluated biomarkers, none of the inflammatory or cardiac biomarkers significantly improved discrimination beyond the baseline clinical model. Because the models were developed and evaluated in the same cohort without resampling-based internal validation, the reported AUC values may be optimistic and should not be interpreted as validated predictive performance.

### 3.11. Sensitivity Analyses

To assess whether the association between inflammatory biomarkers and NOAF varied according to the timing of arrhythmia onset, age-adjusted sensitivity analyses were performed separately for early (<24 h) and delayed (>24 h) NOAF (Table 13). Among patients with early NOAF, log-transformed CRP remained significantly associated with NOAF after adjustment for age (adjusted OR 1.67, 95% CI 1.04–2.68, *p* = 0.035), whereas NLR and SII showed no significant associations.

When delayed NOAF (>24 h) was analyzed separately, log-transformed CRP showed a positive but statistically non-significant association with NOAF (adjusted OR 1.77, 95% CI 0.96–3.28, *p* = 0.067). NLR and SII were also not significantly associated with delayed NOAF.

Given the limited number of events, particularly the 11 delayed NOAF events, the timing-based sensitivity analyses should be interpreted as exploratory and their estimates may be unstable. The direction of the association observed for CRP was broadly similar across the timing-based analyses, whereas the findings for NLR and SII were less consistent; however, the imprecision of these estimates precludes confirmatory conclusions.

In order to examine whether the association between admission CRP and NOAF was influenced by atrial remodeling, a parsimonious sensitivity model including log-transformed admission CRP, age, and left atrial volume was fitted. The results are presented in Table 14. All 100 patients, including 33 with NOAF, were included in this analysis. Admission CRP remained associated with NOAF after adjustment for age and left atrial volume (adjusted OR 1.56, 95% CI 1.02–2.46, *p* = 0.045). The CRP effect estimate was modestly attenuated compared with that observed in the main clinical model incorporating CRP (adjusted OR 1.71, 95% CI 1.08–2.91, *p* = 0.031). Age remained associated with NOAF (adjusted OR 1.09 per year, 95% CI 1.04–1.15, *p* = 0.001), as did left atrial volume (adjusted OR 1.64 per 10 mL increase, 95% CI 1.29–2.20, *p* < 0.001).

## 4. Discussion

The present study examined the relationship between systemic inflammation and the occurrence of new-onset atrial fibrillation during hospitalization for acute coronary syndrome. The main finding was that patients who developed NOAF exhibited a more pronounced inflammatory profile at admission, with significantly higher CRP concentrations and increased composite inflammatory indices. Among the evaluated inflammatory biomarkers, CRP showed the most consistent relationship with NOAF and remained associated with its occurrence in exploratory analyses adjusted for clinically selected covariates. In contrast, NLR and SII lost statistical significance after multivariable adjustment. CRP also showed the greatest numerical improvement in model discrimination when added to the baseline clinical model, although the increase in AUC did not reach statistical significance. Furthermore, sensitivity analyses addressing both arrhythmia timing and potential confounding by age and left atrial volume supported a similar direction of association for CRP, although these findings should be interpreted cautiously because of the limited number of events. The incidence of NOAF in the present cohort (33%) was notably higher than that reported in several published ACS populations, in which newly diagnosed atrial fibrillation has been observed in approximately 10% of patients [19,20]. This discrepancy may reflect differences in patient selection, baseline clinical characteristics, and rhythm surveillance strategies. Notably, our cohort predominantly comprised patients with STEMI (81%), and patients who developed NOAF were older and had a greater burden of cardiovascular comorbidities and structural cardiac abnormalities. Continuous bedside ECG monitoring during coronary care unit hospitalization, followed by 24-h Holter monitoring in patients without previously documented AF, may also have facilitated the identification of arrhythmic episodes. Nevertheless, rhythm monitoring was not continuous throughout the entire hospitalization, and differences in surveillance alone cannot fully explain the higher observed incidence. Given the relatively small, single-center cohort and its specific clinical composition, the 33% incidence should be interpreted cautiously and should not be considered representative of the broader ACS population. Taken together, these findings support a potential role of systemic inflammation in identifying patients with ACS who are more susceptible to developing NOAF during the acute phase of hospitalization.

The association between inflammatory activation and atrial fibrillation has been recognized for several years, and elevated CRP concentrations have been associated with both prevalent and incident AF in different clinical settings. In patients with acute myocardial infarction, Aronson et al. earlier reported an association between elevated CRP and the development of NOAF, supporting the hypothesis that inflammatory activation may contribute to atrial electrical instability during acute ischemic injury [10].

Later evidence has reinforced this relationship, with studies showing that inflammatory biomarkers measured during acute myocardial infarction (AMI) are associated with the occurrence of NOAF [21]. The relevance of inflammatory dynamics has also been demonstrated by Yoshizaki et al., who reported that serial CRP concentrations were significantly higher in patients who developed AF during the early phase of acute myocardial infarction, with CRP levels measured on days 2–4 remaining independently associated with AF occurrence [22]. More recently, patients who developed NOAF during AMI were reported to have particularly high CRP concentrations compared with those with pre-existing AF, further suggesting that acute inflammatory activation may characterize this arrhythmic phenotype [23,24]. Our findings are consistent with these observations, showing that CRP retained a significant association with NOAF after adjustment for clinical covariates, whereas other inflammatory indices did not retain statistical significance.

Several mechanisms may explain the observed relationship between inflammation and NOAF. Acute myocardial ischemia triggers a systemic inflammatory response that involves cytokine activation, endothelial dysfunction, oxidative stress, and changes in autonomic tone. These processes may promote atrial conduction heterogeneity and increase susceptibility to atrial arrhythmogenesis [5]. CRP may, therefore, represent an integrated marker of the inflammatory burden accompanying acute coronary injury rather than a direct mediator of arrhythmia. This distinction is particularly important in an observational study, as the present data support an adjusted association but do not establish a causal relationship. Higher admission CRP concentrations among patients who subsequently developed NOAF indicate that an association was already observable before the first documented arrhythmic episode, but do not establish a temporal or causal relationship. Importantly, admission and NOAF-day CRP concentrations were obtained from separate blood samples in all 33 patients and were not reused or imputed. Nevertheless, among the 22 patients with NOAF onset within 24 h, the interval between measurements was short and 18 patients had the same reported CRP concentration in both samples. Additionally, the sensitivity analysis with a sampling interval longer than 24 h included only 11 patients. Therefore, the observed within-patient CRP changes should be regarded as exploratory and should not be interpreted as evidence that increasing CRP concentrations preceded or triggered NOAF onset.

An intriguing finding was the differential pattern observed across inflammatory markers after multivariable adjustment. NLR and SII were significantly higher in patients who developed NOAF and were associated with NOAF in univariable analyses, but these associations were attenuated after adjustment for clinical covariates [25]. Unlike CRP, which reflects the hepatic acute-phase response, NLR and SII are composite indices derived from circulating blood cell counts and may be influenced by several acute physiological processes, including catecholamine release, stress-related lymphopenia, and changes in circulating neutrophil and platelet populations [26]. Beyond the setting of NOAF, hematological inflammatory indices have also been investigated in patients undergoing coronary artery bypass grafting, particularly in relation to postoperative mortality, further illustrating their potential relevance as readily available markers of inflammatory burden in coronary disease [26,27]. Recent studies have explored composite inflammatory markers and CRP-based indices in relation to NOAF after acute myocardial infarction, supporting the potential relevance of immune-inflammatory imbalance in this setting [28]. In the present cohort, CRP retained a significant association with NOAF after adjustment, whereas NLR and SII did not. This finding may suggest that CRP more closely reflects the systemic inflammatory component associated with NOAF in the acute coronary setting, although this interpretation requires confirmation in larger populations.

Cardiac biomarkers showed a different pattern. NT-proBNP concentrations were numerically higher among patients who subsequently developed NOAF, whereas troponin levels were similar between groups. Previous studies have documented associations between natriuretic peptides and NOAF after myocardial infarction, potentially reflecting increased ventricular and atrial wall stress and hemodynamic impairment [29]. Parashar et al. reported independent associations of both NT-proBNP and hs-CRP with new-onset AF during hospitalization after myocardial infarction [9], while other studies have demonstrated predictive value for BNP in patients with STEMI undergoing primary PCI [30]. In the present study, however, NT-proBNP was not independently associated with NOAF after multivariable adjustment, while troponin showed no significant association. In contrast, CRP retained its association after adjustment for clinical covariates, suggesting a potentially stronger relationship between systemic inflammatory activity and NOAF in this cohort than that observed for markers of myocardial injury or hemodynamic stress.

The clinical and structural characteristics of patients who developed NOAF were also consistent with a more vulnerable atrial substrate. Older age showed a consistent association with NOAF across the adjusted models, while larger left atrial diameter and volume were strongly associated with NOAF in univariable analyses. Previous studies have similarly identified left atrial enlargement and indices of atrial remodeling as factors associated with NOAF after acute myocardial infarction [31]. These observations are biologically plausible, as structural atrial changes may favor re-entry and conduction heterogeneity in the presence of acute triggers such as ischemia and inflammation [32,33]. In the parsimonious sensitivity model accounting for age and left atrial volume, admission CRP remained associated with NOAF, although its effect estimate was modestly attenuated. Left atrial volume also remained strongly associated with NOAF, supporting the importance of the underlying structural atrial substrate.

In a broader biological context, the BETA-MI study by Licordari et al. examined Wnt/β-catenin signaling in patients with acute myocardial infarction, providing additional insight into pathways associated with left ventricular function after STEMI. Although relevant to the complex response to myocardial infarction and cardiac remodeling, these findings do not directly address CRP or NOAF [34].

Patients with NOAF also had a greater angiographic disease burden, particularly a higher prevalence of three-vessel coronary artery disease. More extensive coronary disease may indicate greater ischemic burden and a more pronounced systemic inflammatory response, both of which could increase susceptibility to atrial arrhythmia. Furthermore, 22 of the 33 NOAF episodes occurred after a coronary procedure. This temporal pattern raises the possibility that peri-procedural ischemic, hemodynamic, autonomic, or inflammatory stress contributed to arrhythmia onset in some patients. However, because procedural timing was defined in relation to NOAF onset and was not a baseline exposure applicable in the same manner to the entire cohort, it could not be evaluated as a conventional baseline covariate in the logistic regression model. The observed temporal relationship should therefore not be interpreted as evidence of a causal effect of the coronary procedure. Taken together, these findings suggest that NOAF may reflect the interaction between a pre-existing vulnerable atrial substrate, the severity of coronary disease, systemic inflammation, and acute peri-procedural triggers.

Importantly, elevated CRP concentrations in patients with NOAF may partially reflect the severity of the acute coronary event and the underlying clinical and structural substrate rather than a direct inflammatory contribution to arrhythmia development alone. The greater burden of coronary disease and atrial remodeling observed in these patients may contribute both to systemic inflammatory activation and to increased susceptibility to atrial fibrillation. Although admission CRP remained associated with NOAF in the parsimonious sensitivity model accounting for age and left atrial volume, residual confounding by coronary disease severity, peri-procedural factors, and other unmeasured variables cannot be excluded. Therefore, the observed association should not be interpreted as evidence of a causal relationship between CRP elevation and NOAF.

From a clinical perspective, CRP has several practical advantages as a potential risk-stratification marker. It is affordable, widely available, routinely measured in patients with ACS, and does not require specialized testing. Nevertheless, the potential clinical value of a biomarker also depends on whether it provides additional information beyond established clinical variables. In this regard, the ROC analysis provides an important perspective: although the addition of CRP produced the greatest numerical increase in AUC among the evaluated biomarkers, the improvement over the baseline clinical model was modest and did not reach statistical significance. Therefore, although CRP was associated with NOAF after adjustment for clinical covariates, its addition did not demonstrate a statistically significant improvement in model discrimination (ΔAUC = 0.035; *p* = 0.266). Moreover, because the AUC values were estimated in the same cohort used to fit the models and were not corrected for optimism through bootstrap or another internal-validation procedure, they represent apparent performance and may overestimate discrimination in new patients. These findings should be regarded as exploratory and hypothesis-generating rather than evidence supporting the immediate clinical use of CRP for NOAF prediction. Larger prospective studies with external validation are needed to establish whether CRP provides clinically meaningful incremental predictive value beyond conventional clinical assessment.

### 4.1. Future Perspectives

Early risk stratification for new-onset atrial fibrillation remains a key objective in the management of acute coronary syndrome. The consistent association between CRP and NOAF observed in the present study supports further investigation into the role of systemic inflammation in atrial arrhythmogenesis [34]. Prospective, multicenter studies involving larger patient populations are needed to validate these findings and to determine whether incorporating CRP into dedicated risk-stratification models provides incremental value beyond established clinical and echocardiographic factors. Future research may also benefit from more individualized and dynamic cardiovascular assessment, as evaluation beyond resting conditions has been shown to provide additional clinically relevant information in selected cardiac populations [35].

Serial assessment of inflammatory biomarkers, together with the evaluation of more specific inflammatory mediators, such as interleukin-6 and other cytokines, may also help elucidate the temporal relationship between inflammatory activation and NOAF development [36,37,38]. Beyond its role in NOAF, inflammation has gained increasing attention as a therapeutic target in secondary cardiovascular prevention, as highlighted by Sabouret et al. [39]. However, evidence regarding anti-inflammatory treatment after myocardial infarction remains mixed. While the COLCOT trial demonstrated a reduction in ischemic cardiovascular events with colchicine [40], the CLEAR SYNERGY trial did not show a significant benefit for its primary cardiovascular outcome [41]. Importantly, these findings do not establish that anti-inflammatory therapies prevent NOAF during ACS. Further studies are needed to explore whether targeting inflammatory pathways could influence NOAF occurrence and related clinical outcomes.

### 4.2. Limitations

The findings of the present study should be interpreted in light of several limitations. First, despite its prospective design, this study included a relatively small sample and only 33 NOAF events. Consequently, the events-per-parameter ratio was limited in both the baseline and biomarker-extended models, increasing the risk of overfitting, unstable regression coefficients, imprecise confidence intervals, and optimistic estimates of model discrimination. This concern is especially relevant to the timing-based sensitivity analysis of delayed NOAF, which included only 11 events. Penalized estimation and bootstrap-based optimism correction were not performed, and the models underwent neither resampling-based internal validation nor external validation. Accordingly, the regression and ROC findings should be considered exploratory and hypothesis-generating. Second, the single-center setting may limit the generalizability of the findings to other patient populations. Third, although continuous bedside ECG monitoring was performed during the coronary care unit stay and subsequent 24-h ECG Holter monitoring was performed in patients without previously detected atrial fibrillation, continuous electrocardiographic surveillance was not maintained throughout the entire hospitalization. Variability in the duration of bedside monitoring and the timing of Holter recordings, partly related to device availability, may have influenced the detection of brief or asymptomatic AF episodes and, consequently, the observed incidence of NOAF. Furthermore, although patients with a previously documented history of AF were excluded and sinus rhythm was confirmed on the admission ECG, previously unrecognized paroxysmal AF cannot be entirely ruled out. In addition, left atrial volume was assessed as an absolute value rather than indexed to body surface area, which may have influenced the evaluation of left atrial size across patients with different body sizes. As the data required for indexation were not systematically collected, left atrial volume index could not be calculated retrospectively. Although the parsimonious sensitivity model accounted for age and left atrial volume, residual confounding by coronary disease burden, ACS severity, and peri-procedural factors cannot be excluded. Given the limited number of NOAF events, these factors were not added simultaneously to the model to avoid further overfitting. In addition, procedural timing was defined in relation to NOAF onset rather than as a baseline exposure applicable to the entire cohort and therefore could not be evaluated as a conventional baseline covariate. Although the admission and NOAF-day CRP concentrations were obtained from separate blood samples, the interval between the two measurements varied according to the timing of NOAF onset and was particularly short in patients with arrhythmia occurring within 24 h. This temporal heterogeneity may have influenced the magnitude of the observed within-patient CRP changes. Furthermore, culprit-vessel location was not systematically recorded as a separate study variable, preventing a reliable comparison of culprit-vessel distribution according to NOAF status. Finally, more specific inflammatory mediators, such as interleukin-6, interleukin-1β, and tumor necrosis factor-α, were not available, limiting the ability to characterize specific inflammatory pathways.

## 5. Conclusions

CRP was independently associated with the occurrence of new-onset atrial fibrillation during hospitalization in patients with acute coronary syndrome and emerged as the most consistent inflammatory biomarker evaluated in the present study. Patients who developed NOAF were also characterized by older age, greater left atrial dimensions, and more extensive coronary artery disease. Collectively, these findings support an association between systemic inflammation and NOAF among patients with acute coronary syndrome, but further studies are needed to determine whether the integration of inflammatory biomarkers with established clinical and echocardiographic factors may improve risk stratification for NOAF.

## Figures and Tables

**Table 1:** Incidence, timing, procedural status, and short-term rhythm outcome of New-Onset Atrial Fibrillation.

Variable	Result
No new-onset AF	67 (67.0%)
New-onset AF	33 (33.0%)
New-onset AF <24 h	22 (66.7%)
New-onset AF 24–48 h	7 (21.2%)
New-onset AF >48 h	4 (12.1%)
Postprocedural new-onset AF	22 (66.7%)
Anteprocedural new-onset AF	11 (33.3%)
Heart rate during new-onset AF, bpm	130 [120–140]
Sinus rhythm conversion	30 (90.9%)
No sinus rhythm conversion	3 (9.1%)

Abbreviations: AF—atrial fibrillation; bpm—beats per minute; h—hours; IQR—interquartile range.

**Table 2:** Baseline clinical, echocardiographic, and biochemical characteristics according to new-onset atrial fibrillation.

Variable	No New-Onset AF	New-Onset AF	*p*-Value
Age, years	60 [52–72]	72 [65–77]	<0.001
Male sex	48 (71.6%)	21 (63.6%)	0.416
BMI, kg/m^2^	27.3 [25.5–29.2]	29.0 [26.1–32.7]	0.033
Hypertension	42 (62.7%)	29 (87.9%)	0.009
Diabetes mellitus	21 (31.3%)	12 (36.4%)	0.616
Current smoker	42 (62.7%)	14 (42.4%)	0.055
Dyslipidemia	39 (58.2%)	31 (93.9%)	<0.001
Chronic kidney disease	5 (7.5%)	12 (36.4%)	<0.001
Systolic blood pressure at admission, mmHg	135 [115–150]	130 [110–140]	0.347
Diastolic blood pressure at admission, mmHg	85 [70–90]	80 [67–90]	0.213
Heart rate at admission, bpm	80 [68.5–98.5]	78 [70–98]	0.552
Left ventricular ejection fraction, %	40 [35–45]	35 [30–40]	0.167
Left atrial diameter, cm	3.8 [3.2–4.1]	4.3 [4.2–4.6]	<0.001
Left atrial volume, mL	59 [46.5–75.5]	87 [72–100]	<0.001
Creatinine, mg/dL	1.09 [0.95–1.34]	1.45 [1.15–1.68]	0.002

Abbreviations: BMI—body mass index; bpm—beats per minute; AF—atrial fibrillation; LVEF—left ventricular ejection fraction.

**Table 3:** Acute coronary syndrome phenotype according to new-onset atrial fibrillation.

Variable	No New-Onset AF	New-Onset AF	*p*-Value
ACS type, overall distribution			0.851
STEMI	54 (80.6%)	27 (81.8%)	
NSTEMI	8 (11.9%)	4 (12.1%)	
Unstable angina	5 (7.5%)	2 (6.1%)	

Abbreviations: ACS—acute coronary syndrome; AF—atrial fibrillation; NSTEMI- non-ST-segment elevation myocardial infarction; STEMI—ST-segment elevation myocardial infarction.

**Table 4:** Coronary disease extent, lesion distribution, and revascularization status according to new-onset atrial fibrillation.

Variable	No New-Onset AF	New-Onset AF	*p*-Value
Extent of coronary artery disease			<0.001
Single-vessel disease	31 (46.3%)	10 (30.3%)	
Two-vessel disease	27 (40.3%)	7 (21.2%)	
Three-vessel disease	9 (13.4%)	16 (48.5%)	<0.001
Coronary artery involvement			
Left anterior descending artery (LAD)	52 (77.6%)	24 (72.7%)	0.591
Left circumflex artery (LCx)	22 (32.8%)	16 (48.5%)	0.130
Right coronary artery (RCA)	33 (49.3%)	23 (69.7%)	0.053
Left main coronary artery (LMCA)	1 (1.5%)	4 (12.1%)	0.040
Revascularization strategy			1.000
PCI	66 (98.5%)	32 (97.0%)	
CABG	1 (1.5%)	1 (3.0%)	
Revascularization status			0.067
Complete revascularization	50 (74.6%)	18 (54.5%)	
Incomplete revascularization	17 (25.4%)	15 (45.5%)	

Table note: Data are presented as n (%). The overall distribution of coronary artery disease extent was compared using the Chi-square test. Comparisons of categorical variables between groups were performed using the Chi-square test or Fisher’s exact test, as appropriate. Complete and incomplete revascularization were assessed among patients undergoing coronary revascularization. Abbreviations: AF—atrial fibrillation; CABG—coronary artery bypass grafting; LAD—left anterior descending artery; LCx—left circumflex artery; LMCA—left main coronary artery; PCI—percutaneous coronary intervention; RCA—right coronary artery.

**Table 5:** Cardiac biomarkers according to new-onset atrial fibrillation.

Variable	No New-Onset AF	New-Onset AF	*p*-Value
Troponin	7000 [501–27,347.5]	8673 [1146–20,774]	0.965
NT-proBNP, pg/mL	1800.4 [988.8–5622.9]	3750 [2134.4–7650]	0.067

Abbreviations: AF, atrial fibrillation; IQR, interquartile range; NTproBNP, N-terminal pro-B-type natriuretic peptide.

**Table 6:** Admission inflammatory markers and composite inflammatory indices according to New-Onset Atrial Fibrillation.

Marker	No New-Onset AF	New-Onset AF	*p*-Value
CRP, mg/L	16.9 [7.3–51.1]	57.2 [29.4–105.3]	<0.001
Leukocytes, cells/µL	12,640 [9840–16,360]	12,860 [11,370–15,030]	0.687
Neutrophils, cells/µL	9390 [7125–13,415]	11,120 [8150–12,360]	0.482
Lymphocytes, cells/µL	1680 [1200–2315]	1250 [940–1930]	0.028
Monocytes, cells/µL	630 [505–830]	750 [560–1070]	0.156
Platelets, cells/µL	264,000 [224,500–307,000]	232,000 [205,000–317,000]	0.391
NLR	5.01 [3.29–10.06]	7.87 [5.23–10.23]	0.036
PLR	152.3 [110.0–213.4]	196.8 [137.3–330.3]	0.053
MLR	0.38 [0.26–0.58]	0.60 [0.35–0.84]	0.008
SII, ×10^9^/L	1371.9 [823.0–2863.4]	1972.6 [1276.9–4327.0]	0.040

Table note: Blood cell counts are reported in cells/µL. Because these raw counts were used in the calculation, the resulting SII was divided by 1000 and is reported in ×10^9^/L on the conventional scale used in the literature. Abbreviations: CRP—C-reactive protein; AF—atrial fibrillation; MLR—monocyte-to-lymphocyte ratio; NLR—neutrophil-to-lymphocyte ratio; PLR—platelet-to-lymphocyte ratio; SII—systemic immune-inflammation index.

**Table 7:** Changes in C-reactive protein from hospital admission to new-onset atrial fibrillation.

Variable	Result
Patients included in paired CRP analysis	33
CRP at admission, mg/L	57.2 [29.4–105.3]
CRP on new-onset AF day, mg/L	57.2 [33.0–105.3]
CRP delta, mg/L	0 [0–12.3]
Wilcoxon signed-rank *p*-value	0.020
CRP delta in new-onset AF <24 h, mg/L	0 [0–0]
CRP delta in new-onset AF 24–48 h, mg/L	15.0 [5.7–27.0]
CRP delta in new-onset AF >48 h, mg/L	28.6 [7.5–46.0]
Kruskal–Wallis *p*-value for CRP delta by timing	0.064
CRP delta in anteprocedural/non-postprocedural AF, mg/L	0 [0–0]
CRP delta in postprocedural AF, mg/L	0 [0–14.3]
Mann–Whitney *p*-value for CRP delta by postprocedural status	0.163
Sensitivity analysis (NOAF onset >24 h)	
Patients included in sensitivity analysis	11
CRP at admission, mg/L	72.7 [26.3–134.7]
CRP on new-onset AF day, mg/L	85.0 [52.7–144.5]
CRP delta, mg/L	15.0 [5.7–31.6]
Wilcoxon signed-rank *p*-value	0.049

Table note: Data are presented as median [interquartile range]. CRP delta (ΔCRP) was defined as the difference between CRP measured from a separate blood sample obtained on the day of the first documented NOAF episode and CRP measured at hospital admission. Two distinct blood samples were available for all 33 patients with NOAF. Among the 22 patients with NOAF onset within 24 h, the admission sample preceded the first documented AF episode in every case; the two separate samples yielded the same reported CRP concentration in 18 patients and different concentrations in four patients. Paired comparisons were performed using the Wilcoxon signed-rank test. Differences in ΔCRP across NOAF timing categories were assessed using the Kruskal–Wallis test, whereas comparisons according to procedural status were performed using the Mann–Whitney U test. The sensitivity analysis was restricted to patients with NOAF onset more than 24 h after admission. Abbreviations: CRP, C-reactive protein; AF, atrial fibrillation; NOAF, new-onset atrial fibrillation.

**Table 8:** Univariable logistic regression analysis for predictors of new-onset atrial fibrillation.

Domain	Predictor	OR	95% CI	*p*-Value
Clinical	Age, per year	1.08	1.04–1.13	<0.001
Clinical	Male sex	0.69	0.29–1.70	0.417
Clinical	BMI, per kg/m^2^	1.17	1.04–1.33	0.013
Clinical	Hypertension	4.32	1.48–15.82	0.013
Clinical	Dyslipidemia	11.13	3.02–72.28	0.002
Clinical	Chronic kidney disease	7.09	2.34–24.52	<0.001
Echocardiography	LVEF, per 1% increase	0.97	0.92–1.02	0.225
Echocardiography	Left atrial diameter, per cm	10.03	3.74–33.53	<0.001
Echocardiography	Left atrial volume, per mL	1.04	1.02–1.07	<0.001
Biochemical	Creatinine, per mg/dL	1.71	0.97–3.15	0.069
Inflammatory	CRP (log-transformed)	1.91	1.31–2.91	0.001
Inflammatory	NLR (log-transformed)	2.13	1.14–4.19	0.021
Inflammatory	MLR	1.77	0.96–3.79	0.093
Inflammatory	SII (×10^9^/L; log-transformed)	1.74	1.07–2.95	0.031
Cardiac biomarkers	Troponin (log-transformed)	1.02	0.86–1.22	0.843
Cardiac biomarkers	NT-proBNP (log-transformed)	1.35	0.93–1.98	0.118
ACS phenotype	STEMI	0.81	0.29–2.40	0.718
Angiography	Three-vessel disease	6.07	2.29–16.09	<0.001
Angiography	RCA lesion	2.37	1.00–5.92	0.056
Angiography	Left main lesion	9.10	1.28–182.29	0.053

Table note: Separate univariable logistic regression models were fitted for each candidate predictor, with new-onset AF as the dependent variable. Results are presented as odds ratios (ORs) with 95% confidence intervals (CIs). Continuous variables are expressed per one-unit increase unless otherwise specified. CRP, NLR, SII, troponin, and NT-proBNP values were log-transformed using the log1p function before analysis. Abbreviations: RCA—right coronary artery; ACS—acute coronary syndrome; BMI—body mass index; CI—confidence interval; CRP—C-reactive protein; AF—atrial fibrillation; LVEF—left ventricular ejection fraction; MLR—monocyte-to-lymphocyte ratio; NLR—neutrophil-to-lymphocyte ratio; NT-proBNP—N-terminal pro-B-type natriuretic peptide; OR—odds ratio; SII—systemic immune-inflammation index; STEMI—ST-segment elevation myocardial infarction.

**Table 9:** Baseline clinical model for new-onset atrial fibrillation.

Variable	Adjusted OR	95% CI	*p*-Value
Age, per year	1.08	1.03–1.14	0.003
Male sex	1.24	0.41–3.87	0.706
LVEF, per 1% increase	1.00	0.94–1.07	0.908
Creatinine, per mg/dL	1.33	0.68–2.56	0.395
STEMI	1.08	0.34–3.59	0.897

Table note: Multivariable logistic regression was performed with new-onset AF as the dependent variable. The baseline model included age, male sex, left ventricular ejection fraction, creatinine, and STEMI. Results are presented as adjusted odds ratios (ORs) with 95% confidence intervals (CIs). Continuous variables are expressed per one-unit increase unless otherwise specified. Abbreviations: AF—atrial fibrillation; CI—confidence interval; LVEF—left ventricular ejection fraction; OR—odds ratio; STEMI—ST-segment elevation myocardial infarction.

**Table 10:** Clinical models incorporating inflammatory biomarkers for new-onset atrial fibrillation.

Model	Marker Added	Adjusted OR	95% CI	*p*-Value	AIC
Clinical + CRP	CRP, log-transformed	1.71	1.08–2.91	0.031	118.5
Clinical + NLR	NLR, log-transformed	1.75	0.84–3.92	0.150	121.6
Clinical + SII	SII (×10^9^/L; log-transformed)	1.52	0.84–2.88	0.176	121.9

Table note: Each inflammatory biomarker was added separately to the baseline clinical model, which included age, male sex, LVEF, creatinine, and STEMI. Multivariable logistic regression was performed with new-onset AF as the dependent variable. Results are presented as adjusted odds ratios (ORs) with 95% confidence intervals (CIs). CRP, NLR, and SII were log-transformed using the log1p function before analysis. Abbreviations: AIC—Akaike information criterion; AF—atrial fibrillation; CI- confidence interval; CRP—C-reactive protein; LVEF—left ventricular ejection fraction; NLR—neutrophil-to-lymphocyte ratio; OR—odds ratio; SII—systemic immune-inflammation index; STEMI—ST-segment elevation myocardial infarction.

**Table 11:** Cardiac biomarker-adjusted model for new-onset atrial fibrillation.

Variable	Adjusted OR	95% CI	*p*-Value
Age, per year	1.08	1.04–1.14	<0.001
Male sex	1.42	0.50–4.32	0.519
LVEF, per 1% increase	1.00	0.93–1.06	0.912
NT-proBNP, log-transformed	1.17	0.73–1.89	0.504
Troponin, log-transformed	0.99	0.81–1.23	0.951

Table note: Multivariable logistic regression was performed with new-onset AF as the dependent variable. The model included age, male sex, LVEF, log-transformed NT-proBNP, and log-transformed troponin. Results are presented as adjusted odds ratios (ORs) with 95% confidence intervals (CIs). NT-proBNP and troponin were log-transformed using the log1p function before analysis. Abbreviations: AF, atrial fibrillation; CI, confidence interval; LVEF, left ventricular ejection fraction; NT-proBNP, N-terminal pro-B-type natriuretic peptide; OR, odds ratio.

**Table 12:** Discriminative Performance of Clinical, Inflammatory, and Cardiac Biomarker Models for New-Onset Atrial Fibrillation.

Model	AUC	95% CI	*p*-Value vs. Clinical Model
Clinical model	0.753	0.656–0.849	Reference
Clinical + CRP	0.788	0.698–0.877	0.266
Clinical + NLR	0.763	0.668–0.857	0.537
Clinical + SII	0.758	0.662–0.853	0.765
Clinical + NT-proBNP	0.753	0.657–0.849	0.685
Clinical + Troponin	0.757	0.662–0.852	0.403

Table note: Area under the receiver operating characteristic curve values were calculated for each multivariable logistic regression model. The baseline clinical model included age, male sex, left ventricular ejection fraction, creatinine, and STEMI. Each extended model incorporated one biomarker separately into the baseline clinical model. Differences in AUC were assessed using DeLong’s test. Abbreviations: AUC—area under the receiver operating characteristic curve; CI—confidence interval; CRP—C-reactive protein; LVEF—left ventricular ejection fraction; NLR—neutrophil-to-lymphocyte ratio; NT-proBNP—N-terminal pro-B-type natriuretic peptide; SII—systemic immune-inflammation index; STEMI—ST-segment elevation myocardial infarction.

**Table 13:** Sensitivity analyses for inflammatory predictors of new-onset atrial fibrillation.

Sensitivity Analysis	Marker	N	Events	Adjusted OR	95% CI	*p*-Value
Early new-onset AF <24 h	CRP, log-transformed	89	22	1.67	1.04–2.68	0.035
Early new-onset AF <24 h	NLR, log-transformed	89	22	1.93	0.87–4.30	0.106
Early new-onset AF <24 h	SII (×10^9^/L; log-transformed)	89	22	1.63	0.88–2.99	0.117
Delayed new-onset AF >24 h	CRP, log-transformed	78	11	1.77	0.96–3.28	0.067
Delayed new-onset AF >24 h	NLR, log-transformed	78	11	1.87	0.72–4.85	0.196
Delayed new-onset AF >24 h	SII (×10^9^/L; log-transformed)	78	11	1.55	0.72–3.33	0.261

Table note: Age-adjusted logistic regression models were fitted separately for each inflammatory biomarker. Early new-onset AF was defined as AF occurring within 24 h after admission, whereas delayed new-onset AF was defined as AF occurring more than 24 h after admission. For the analysis of early new-onset AF, patients with delayed AF were excluded, and patients without new-onset AF served as the reference group. Conversely, for the analysis of delayed new-onset AF, patients with early AF were excluded, while patients without new-onset AF served as the reference group. CRP, NLR, and SII were log-transformed using the log1p function before analysis. Results are presented as adjusted odds ratios (ORs) with 95% confidence intervals (CIs). Confidence intervals and two-sided *p* values were calculated using the Wald method. Abbreviations: CI—confidence interval; CRP—C-reactive protein; AF—atrial fibrillation; NLR—neutrophil-to-lymphocyte ratio; OR—odds ratio; SII—systemic immune-inflammation index.

**Table 14:** Parsimonious sensitivity model accounting for left atrial volume.

Variable	Adjusted OR	95% CI	*p*-Value
CRP at admission, log-transformed	1.56	1.02–2.46	0.045
Age, per year	1.09	1.04–1.15	0.001
Left atrial volume, per 10 mL increase	1.64	1.29–2.20	<0.001

Table note: A parsimonious multivariable logistic regression model was fitted with new-onset atrial fibrillation as the dependent variable. The model included log1p-transformed admission CRP, age, and left atrial volume. Left atrial volume was expressed per 10 mL increase. All 100 patients were included, of whom 33 developed new-onset atrial fibrillation. Results are presented as adjusted odds ratios (ORs) with 95% confidence intervals (CIs). Abbreviations: CI, confidence interval; CRP, C-reactive protein; NOAF, new-onset atrial fibrillation; OR, odds ratio.

## Data Availability

Data are available upon request due to restrictions (privacy and ethical reasons).
